# Hypoxia Associated Proteolytic Processing of OS-9 by the Metalloproteinase Meprin *β*

**DOI:** 10.1155/2016/2851803

**Published:** 2016-07-13

**Authors:** Barry Lee Martin, Sabena Michelle Conley, Regine Simone Harris, Corshe Devon Stanley, Jean-Marie Vianney Niyitegeka, Elimelda Moige Ongeri

**Affiliations:** Department of Biology, North Carolina A&T State University, Greensboro, NC 27411, USA

## Abstract

Meprin metalloproteases play a role in the pathology of ischemia/reperfusion- (IR-) induced renal injury. The endoplasmic reticulum-associated protein, osteosarcoma-9 (OS-9), has been shown to interact with the carboxyl-terminal tail of meprin *β*. More importantly, OS-9 interacts with the hypoxia inducible factor-1*α* (HIF-1*α*) and the prolyl-hydroxylase, proteins which mediate the cell's response to hypoxia. To determine if OS-9 is a meprin substrate, kidney proteins from meprin *αβ* knockout mice (*αβ*KO) (which lack endogenous meprins) and purified human OS-9 were incubated with activated forms of meprin A and meprin B, and Western blot analysis was used to evaluate proteolytic processing of OS-9. Fragmentation of OS-9 was observed in reactions with meprin B, but not meprin A. To determine whether meprin B cleaves OS-9* in vivo*, wild-type (WT) and meprin *αβ*KO mice were subjected to IR-induced renal injury. Fragmentation of OS-9 was observed in kidney proteins from WT mice subjected to IR, but not in meprin *αβ*KO counterparts. Transfection of kidney cells (MDCK and HEK293) with meprin *β* cDNA prevented accumulation of OS-9 following exposure to the hypoxia mimic, CoCl_2_. These data suggest that meprin *β* interaction with OS-9 plays a role in the hypoxia response associated with IR-induced renal injury.

## 1. Introduction

Meprins are zinc metalloproteases that are most abundantly expressed in the brush-border membranes (BBMs) of proximal kidney tubules. Several studies have demonstrated a role for meprins in the pathology of ischemia/reperfusion (IR) induced renal injury. Pretreatment of mice with the meprin inhibitor, actinonin, and disruption of the meprin *β* gene both protect mice against IR-induced renal injury [1–3], suggesting that presence of meprins enhances acute kidney injury. However, the mechanisms by which meprins modulate kidney injury in IR are not fully understood. Meprins consist of two subunits, *α* and *β*, resulting in two protein isoforms. Meprin A is a homooligomer of *α*-*α* subunits or a heterodimer of *α*-*β* subunits, while meprin B is a homooligomer of *β*-*β* subunits. While meprin A and meprin B are highly similar and have several shared substrates, there are unique substrates specific for each isoform. Identifying meprin substrates in the kidney has provided insights on how meprins modulate kidney injury in IR. Based on the currently known substrates, meprins could enhance kidney injury via proteolytic processing/degrading of cytoskeletal proteins (e.g., villin and actin) [4] and tight junction proteins (e.g., occludin and E-cadherin) [5, 6]. In IR, meprins have been shown to be redistributed from the BBM to the cytosol and basolateral membranes [1]. This redistribution brings meprins in close proximity to proteins in other cell compartments that could be proteolytically processed and thus impact the cellular response to IR. Meprin targets in the cytosol and basement membrane include cell signaling molecules (e.g., the catalytic subunit of protein kinase A, PKA C) [7, 8] and the extracellular matrix (ECM) proteins (e.g., laminin, fibronectin, nidogen-1, and collagen) [9–12]. The injury associated with IR is due, in large part, to reduced oxygen delivery to renal tissue. The normal response to hypoxia is mediated by hypoxia response genes, which include hypoxia inducible factors (HIFs), for example, HIF-1*α* and HIF-2*α*. HIF-1*α* and HIF-2*α* have cell-type specific effects on gene expression [13]. In the kidney, HIF-1*α* is the predominant form in epithelial cells, while HIF-2*α* is predominant in interstitial fibroblast and endothelial cells [14]. HIF-1*α* target genes encode proteins that enable cells to survive oxygen deprivation by providing oxygen-independent means of ATP production such as glucose transporters and glycolytic enzymes or by inhibiting hypoxia-induced apoptosis for example, through survival factors like insulin-like growth factor 2 (IGF2). Some target gene products increase tissue oxygen delivery by stimulating angiogenesis or erythropoiesis.

Osteosarcoma-9 (OS-9) is a ubiquitously expressed endoplasmic reticulum-associated protein. Studies with a yeast two-hybrid system showed that OS-9 interacts with the carboxyl-terminal tail of meprin *β* [15]. This interaction is significant because OS-9 has also been shown to interact with HIF-1*α* and prolyl hydroxylase [16], proteins which mediate the cell's response to changes in oxygen concentration. Although OS-9 is localized in the endoplasmic reticulum, it is present in both nuclear and cytoplasmic protein extracts, suggesting that it could traffic HIF proteins between the nucleus and the ER during the hypoxic response [14]. The current study was conducted to determine if OS-9 is a meprin substrate and whether there is a correlation between meprin *β* expression and proteolytic processing of OS-9* in vivo *and* in vitro*. The findings will provide new insights on how meprins modulate kidney injury under hypoxic conditions such as IR-induced acute kidney injury.

## 2. Materials and Methods

### 2.1. Experimental Animals

Twelve-week-old wild-type and meprin *αβ* double knockout (*αβ*KO) mice on a C57BL/6 background were used for these studies. The mice were housed at the North Carolina A&T State University Laboratory Animal Resource Unit (LARU), Greensboro, North Carolina. All the mice were kept under a 12 : 12 light : dark cycle and provided rodent chow and fresh water ad libitum. The animal procedures used were approved by the North Carolina A&T State University Institutional Animal Care and Use Committee (IACUC).

### 2.2. Induction of Ischemia Reperfusion

Ischemia reperfusion was induced in WT and meprin *αβ*KO mice (*n* = 6 for each genotype) by bilateral clamping of the renal pedicle for 26 minutes followed by reperfusion for 6 h or 24 h as previously described [1, 4]. Control mice (*n* = 4 for each genotype) were sham-operated without clamping the renal pedicle. The mice were sacrificed at 6 h or 24 h after IR by using isoflurane asphyxiation followed by cervical dislocation. The kidneys were excised and decapsulated. Sections of the kidney were fixed in Carnoy's fixative (60% ethanol, 30% formalin, and 10% acetic acid) overnight and then transferred to 70% ethanol. The kidney tissues were subsequently paraffin embedded at the Penn State Hershey Histology Core Laboratories. The remaining kidney tissues were wrapped in aluminum foil, snap-frozen in liquid nitrogen, and stored at −80°C until used for protein extraction. To confirm kidney injury, the levels of blood urea nitrogen (BUN) were determined using BUN slides (Cat. # 1532332, Ortho-Clinical Diagnostics, Rochester NY) read on a Vitro DT60 II analyzer (Ortho-Clinical Diagnostics).

### 2.3. Kidney Protein Fractionation

The kidney tissues were thawed on ice and homogenized in ice-cold EDTA-free lysis buffer with protease inhibitors. Proteins were fractionated by differential centrifugation as previously described [4, 7, 17] to obtain a BBM- and cytosolic-enriched protein fractions. Some kidney tissues were extracted in RIPA buffer without any fractionation (herein referred to as total protein). Extracted kidney proteins were aliquoted and stored at −80°C until used for Western blot analysis.

### 2.4. Localization of OS-9 in Kidney Tissue

**Figure 1:**
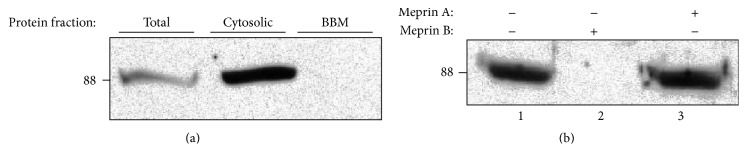
Immunoblot of OS-9 in kidney proteins. Meprin B degrades OS-9 present in kidney proteins. Kidney proteins were fractionated by differential centrifugation into BBM- and cytosolic-enriched fractions. Portions of the kidney tissue were homogenized in RIPA buffer to obtain nonfractionated total kidney proteins. 60 *μ*g proteins from each fraction were loaded onto 10% gels and analyzed by Western blot analysis. (a) OS-9 was detected in total kidney proteins and cytosolic-enriched protein fraction, but not in BBM-enriched protein fractions. (b) Cytosolic-enriched kidney proteins were incubated with Tris buffer without meprins (lane 1), activated meprin B (lane 2), or activated homomeric meprin A (lane 3). Degradation of OS-9 was observed in reactions with activated meprin B, but not meprin A or Tris buffer without meprins.

We first determined the kidney protein fraction that had the most abundant OS-9 levels by loading 60 *μ*g of total (nonfractionated kidney proteins), cytosolic-, and BBM-enriched kidney proteins onto 10% SDS-PAGE gels. The proteins were transferred to nitrocellulose membranes and Western blot analysis was used to evaluate levels of OS-9. Our data showed that OS-9 proteins were most abundant in the cytosolic-enriched protein fraction (Figure 1(a)). No OS-9 was detected in the BBM-enriched fraction. For this reason, subsequent analysis utilized cytosolic-enriched protein fractions.

### 2.5. Determining Whether Meprins Proteolytically Process OS-9 Present in Kidney Proteins

To determine whether meprins are capable of proteolytically processing OS-9 present in kidney tissue, activated purified forms of recombinant homomeric meprin A (*α*-*α*) and meprin B (*β*-*β*) were incubated with 60 *μ*g of cytosolic-enriched kidney proteins from meprin *αβ* double KO mice (which lack endogenous meprins) in Tris buffer (20 mM Tris and 150 mM NaCl, pH 7.5) at 37°C for 4 h. The recombinant homomeric meprin A and meprin B used were purified from stably transfected human embryonic kidney (HEK293) cells and activated using limited trypsin proteolysis as previously described [4, 18]. Briefly, meprins were incubated with trypsin (1 : 20 ratio) in 20 mM Tris, 150 mM NaCl, pH 7.5, at 37°C for 30 min (meprin A) or 1 h (meprin B). The activation was stopped by adding soy bean trypsin inhibitor (STI). The trypsin and STI were removed by running the reaction mixtures through a Sephadex G25 column. Meprin activity was measured by using the substrate azocasein (Sigma-Aldrich, MI). Product fluorescence was measured with a Hitachi F2000 fluorescence spectrophotometer using an excitation wavelength of 320 nm and an emission wavelength of 417 nm. To confirm that the cleavage was meprin B-specific, Tris buffer and latent forms of meprin A and meprin B that were not trypsin-activated were used as negative controls. Additional negative controls used meprin B which was preincubated with 30 *μ*M of the meprin inhibitor, actinonin for 1 h. The products obtained from coincubating meprins with protein lysates were electrophoretically separated and Western blot analysis with anti-OS-9 specific antibodies (Cat. # TA301503, Origene, Rockville MD) was used to evaluate for degradation/fragmentation of OS-9.

### 2.6. Confirming That OS-9 Degradation Is Meprin-Specific

Our preliminary data showed that meprins cleave OS-9 present in kidney proteins. To confirm that degradation of OS-9 was meprin-specific, we incubated purified human OS-9 (Cat. # TP316820, Origene, Rockville, MD) with activated forms of meprin A and meprin B and used Western blot analysis to evaluate protein fragmentation/degradation. To this end, 4 nM activated homomeric meprin A (*α*-*α*) and 4 nM meprin B (*β*-*β*) were incubated with 92 nM purified OS-9 in a total reaction volume of 50–65 *μ*L. Equal volumes of reactants (7 *μ*L) were taken out at 0, 0.25, 0.5, 1, 2, 3, and 4 h. Additional incubations were done for shorter time points totaling 1 h, with samples being taken out at 5 minute intervals. Proteolysis was stopped by addition of SDS sample buffer followed by boiling for 5 minutes. Control reactions were incubated with Tris buffer without meprins. The proteins were electrophoretically separated on 10% SDS gels, and Western blot analysis with anti-OS-9 specific antibodies (Origene, Rockville, MD) was used to evaluate changes in OS-9 levels and fragmentation over time.

### 2.7. Determining Whether Meprins Cleave/Degrade OS-9* In Vivo*

Because OS-9 has been shown to interact with the hypoxia response factor, HIF-1*α*, we chose to work with an* in vivo* hypoxia model (ischemia/reperfusion) which causes acute kidney injury. To evaluate meprin degradation of OS-9* in vivo*, cytosolic-enriched kidney protein fractions from WT and meprin *αβ*KO mice subjected to IR were evaluated for fragmentation using Western blot. The levels of OS-9 in individual protein samples from sham-operated control kidneys and kidneys subjected to 6 h IR were quantified by optic densitometry using Bio-Rad's GS800 calibrated densitometer.

### 2.8. Generation of Meprin *β* cDNA Construct and Transfection of Madin-Darby Canine Kidney (MDCK) Cells

To determine whether meprin B interacts with OS-9* in vitro*, MDCK cells were transfected with a meprin *β* cDNA construct using the Lipofectamine method as previously described [19–21]. The cDNA construct sequence was analyzed at the Penn State Hershey genomic Core Laboratories and the sequence confirmed using Blast alignment before transfecting the cells. Stably transfected cell lines were established using hygromycin B supplemented Dulbecco's modified essential medium (DMEM). Western blot analysis and immunofluorescence staining were used to confirm expression of meprin B proteins by the transfected MDCK cells.

### 2.9. Cell Culture and Induction of Hypoxia* In Vitro*

Meprin *β* transfected and mock transfected control MDCK cells were seeded at 1 × 10^5^ in 100 mm dishes and cultured in DMEM supplemented with 10% FBS and antibiotics/antimycotics at 37°C and 5% CO_2_ until they were 80–90% confluent. Meprin *β* transfected HEK293 cells (a gift from Dr. Judith Bond, Penn State College of Medicine, Hershey, PA) were also used. The HEK293 cells were similarly cultured in MEM media supplemented with 10% FBS and antibiotics/antimycotics. To induce hypoxia, the cells were serum-starved by overnight incubation in serum-free media supplemented with 0.1% BSA. The cells were then exposed to 125 *μ*M cobalt chloride (CoCl_2_), a hypoxia mimic for 0–3 h. Cells for immunofluorescence staining were cultured in 8-well slide chambers and similarly exposed to 125 *μ*M CoCl_2_.

### 2.10. Extraction and Fractionation of Proteins in Cell Lysates

Cell protein lysates were fractionated into cytosolic-, nuclear-, and membrane-enriched fractions using previously described protocols [5, 20]. All cell lysis buffers used were supplemented with protease inhibitors with EDTA (Roche Diagnostics, Indianapolis, IN). Bio-Rad's protein reagent (Cat. # 500-0006) was used to determine the protein concentrations. The proteins were stored at −20°C until used for Western blot analysis.

### 2.11. Western Blot Analysis

Western blot analysis was used to confirm meprin B protein expression by the transfected MDCK cells, to evaluate the levels of HIF-1*α* and OS-9 in kidney proteins and MDCK protein lysates, and for evaluating fragmentation and/or degradation of OS-9. The protocols used were as previously described [4, 7]. To confirm that CoCl_2_ treatment induced hypoxia, nuclear-enriched proteins were probed for HIF-1*α* by incubation in anti-HIF-1*α* antibody (mouse monoclonal, Cat. # ab16066, Abcam, Cambridge, UK), diluted 1 : 1000 in TBS-T. To probe for OS-9, cytosolic- and nuclear-enriched protein fractions were incubated in rabbit polyclonal anti-OS-9 antibodies (Cat. # TA301503, Origene, Rockville, MD), diluted 1 : 1000 in TBS-T. The membranes were exposed to chemiluminescence substrate (West Pico, Cat. # 34080, Thermo Fisher, Pittsburg, PA) and the X-ray film developed. The intensities of the protein bands on the X-ray film were quantified by optic densitometry using Bio-Rad's GS800 calibrated densitometer and Quantity One software. The levels of tubulin were used as a loading control for determining the relative ODs for OS-9 and HIF-1*α*.

### 2.12. Immunofluorescence Staining and Confocal Microscopy

Immunofluorescence staining was used to evaluate protein expression for HIF-1*α* and OS-9 in cultured cells exposed to CoCl_2_. The MDCK cells were cultured in slide chambers until 80–90% confluent and exposed to CoCl_2_ as described above. The cells were fixed by heating at 70°C for 10 minutes in freshly made 1% paraformaldehyde. The cells were then permeabilized in 0.2% triton-X 100 in PBS for 10 min at room temperature (RT). Nonspecific binding sites were blocked in 5% normal goat serum in PBS with 0.1% triton-X-100 for 1 h at room temperature. This was followed by incubation in primary antibodies at RT for 1 h or overnight at 4°C in a humidified chamber. The antibodies used were rabbit polyclonal anti-meprin B (HMC77, a gift from Dr. Judith Bond, Pennsylvania State University, Hershey, PA) diluted 1 : 400, mouse monoclonal anti-HIF1*α* (Abcam, Cambridge, UK) diluted 1 : 200, and rabbit polyclonal anti-OS-9 (Origene, Rockville, MD) diluted 1 : 200. Secondary antibodies used were anti-rabbit Alexa fluor488 (Cat. # 4412, Cell Signaling, Boston, MA) diluted 1 : 1000 or anti-mouse Alexa fluor555 (Cat. # 4409, Cell Signaling, Boston MA). DAPI was used for nuclei staining. The slide sections were imaged using a confocal microscope (Carl Zeiss Microscopy, LLC, Thornwood, NY) with AxioVision software.

### 2.13. Statistical Analysis

All Western blots were repeated at least 3 times. Graph Pad Prism software tools were used for statistical analysis. Two-way ANOVA with pairwise comparisons was employed to evaluate protein levels following quantitation of the protein bands by optic densitometry. *P* values ≤ 0.05 were considered significant.

## 3. Results

### 3.1. Proteolytic Processing of OS-9 by Meprins Is Isoform-Specific

**Figure 2:**
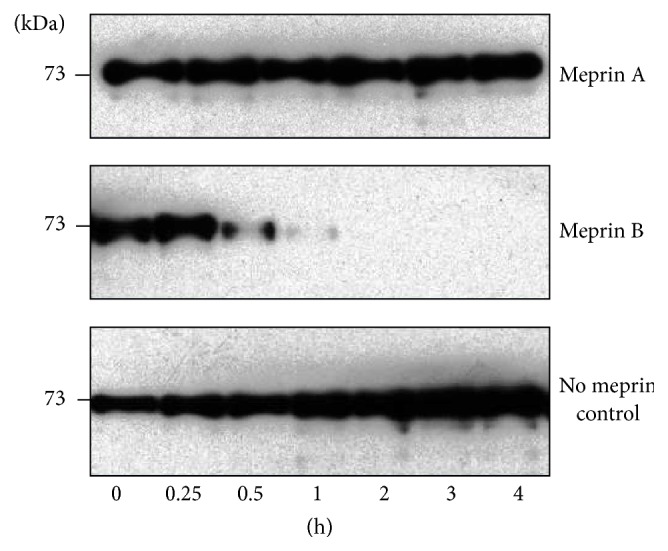
Meprin B degrades purified recombinant human OS-9. Purified recombinant OS-9 was incubated with activated forms of homomeric meprin A and meprin B for 0–4 h. Western blot analysis was used to evaluate for degradation or fragmentation of OS-9. The levels of OS-9 decreased in a time-dependent manner in reactions with activated meprin B, but not in reactions with activated meprin A or Tris buffer controls without meprins.

**Figure 3:**
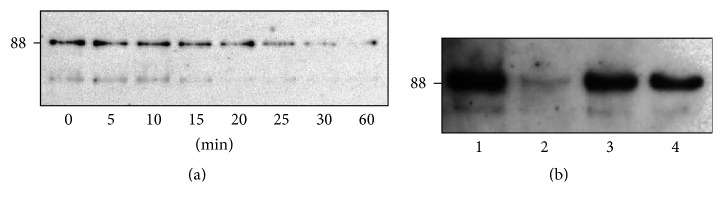
Degradation of OS-9 is meprin B-specific. (a) Time-depended degradation of purified OS-9 incubated with activated purified recombinant meprin B. (b) Degradation of OS-9 was blocked by preincubation with the meprin inhibitor, actinonin. Purified OS-9 was incubated with Tris buffer only (lane 1), activated meprin B (lane 2), latent meprin B (lane 3), and activated meprin B preincubated with the meprin inhibitor actinonin (lane 4). Degradation of OS-9 was not observed with the latent form of meprin B. Degradation of OS-9 was also blocked by preincubation of meprin B with the meprin inhibitor, actinonin.

Western blot analysis of mouse kidney proteins detected OS-9 (~88 kDa) predominantly in the cytosolic-enriched kidney protein fractions (Figure 1(a)). Incubation of cytosolic-enriched kidney proteins from meprin *αβ*KO mice with activated meprin B resulted in degradation of OS-9 present in the kidney proteins. This degradation was not observed when proteins were incubated with activated meprin A (Figure 1(b)). To confirm that the degradation of OS-9 was meprin-specific, activated forms of recombinant homomeric mouse meprin A (*α*-*α*) and rat meprin B (*β*-*β*) were incubated with purified human OS-9 for 0–4 h at 37°C. A time-dependent degradation of the purified OS-9 was observed in reactions with activated meprin B, but not in proteins incubated with activated meprin A or Tris buffer without meprins (Figures 2 and 3). Degradation of OS-9 was not observed in control reactions incubated with latent meprin B or reactions in which activated meprin B was preincubated with the meprin inhibitor, actinonin (Figure 3(b)). However, we did not observe accumulation of OS-9 intermediate fragments when purified OS-9 was incubated with meprin B.

### 3.2. Ischemia Reperfusion Was Associated with* In Vivo* Fragmentation of OS-9 in Meprin-Expressing Kidneys

**Figure 4:**
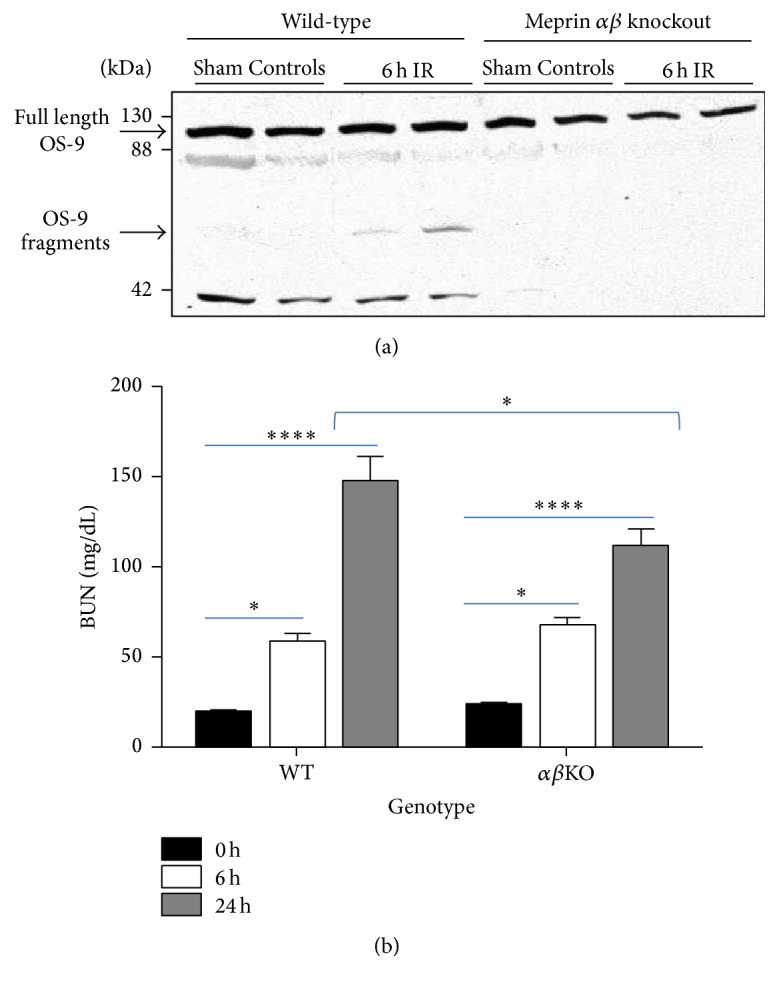
Meprin B cleaves kidney OS-9 in ischemia reperfusion, immunoblot for OS-9 in individual kidney samples. (a) Representative immunoblot for OS-9 in cytosolic-enriched kidney protein fractions from mice subjected to IR-induced renal injury and sham-operated controls. 12-week-old wild-type (WT) and meprin *αβ* knockout (*αβ*KO) mice were subjected to IR-induced renal injury by clamping of renal pedicle for 26 minutes followed by 6 h of reperfusion. Western blot analysis was used to evaluate fragmentation of OS-9 in cytosolic-enriched kidney protein fractions. Fragmentation of OS-9 was observed in kidney proteins from WT mice subjected to IR, but not in sham-operated WT controls or meprin *αβ*KO mice subjected to IR. A unique (~60 KDa) OS-9 fragment was observed in protein samples from WT kidneys subjected to IR. (b) Blood urea nitrogen (BUN) levels were used as a biochemical assessment of kidney injury. ^*∗*^ *P* ≤ 0.05; ^*∗∗∗∗*^ *P* ≤ 0.0001.

To determine whether meprins proteolytically process OS-9 present in kidney tissue* in vivo*, Western blot analysis was used to evaluate fragmentation of OS-9 in cytosolic-enriched kidney proteins from WT and meprin *αβ*KO mice subjected to IR-induced acute kidney injury. Kidney injury was first confirmed by assays for blood urea nitrogen (BUN) (Figure 4(b)). The BUN levels were significantly higher in mice subjected to IR injury when compared to sham-operated controls at 6 and 24 h after IR (*P* ≤ 0.05 and *P* ≤ 0.0001, resp.). BUN levels at 24 for WT were also significantly higher than for the meprin *αβ*KO at the same time point (*P* ≤ 0.05). The pattern for markers of kidney injury was consistent with data from previous studies where the plasma creatinine levels were significantly higher in WT mice when compared to meprin *β* knockout counterparts at 6 and 24 h after IR [1]. The Western blot data showed fragmentation of the OS-9 present in samples from WT mouse kidneys (which express normal levels of meprin A and meprin B) at 6 h after IR. The IR-associated fragmentation produced a unique ~60 kDa OS-9 fragment in proteins from WT kidneys at 6 h after IR. This fragmentation was not observed in proteins from meprin *αβ*KO kidneys (which are deficient in meprin A and meprin B) subjected to IR or WT control kidneys not subjected to IR (Figure 4(a)). The data suggest that the presence of both meprins and hypoxia/ATP-depletion associated with IR plays a role in the cleavage of OS-9 to produce the 60 kDa fragment. A second fragment (~37 kDa) was also detected in the meprin-expressing kidneys. However, its presence did not correlate to IR-induced kidney injury.

### 3.3. Hypoxia-Induced Degradation of OS-9 in Meprin *β* Expressing Kidney Cell Lines

**Figure 5:**
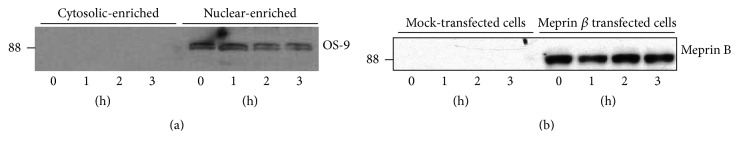
Immunoblots for OS-9 and meprin B in MDCK cells exposed to CoCl_2_. (a) OS-9 in different cell protein fractions. Protein lysates from MDCK cells were fractionated into a nuclear- and cytosolic-enriched fractions and Western blot analysis used to evaluate the levels of OS-9 in the two fractions. OS-9 was predominantly present in the nuclear-enriched fraction, and the levels decreased following exposure to CoCl_2_. (b) Meprin B in meprin *β* transfected and mock transfected control MDCK cells. The Lipofectamine method coupled with hygromycin supplemented media was used to generate MDCK cells stably transfected with meprin *β* cDNA. Western blot analysis showed meprin B expression only in the MDCK cells transfected with meprin *β* cDNA and not the mock transfected control cells. CoCl_2_ exposure did not alter meprin B protein expression.

**Figure 6:**
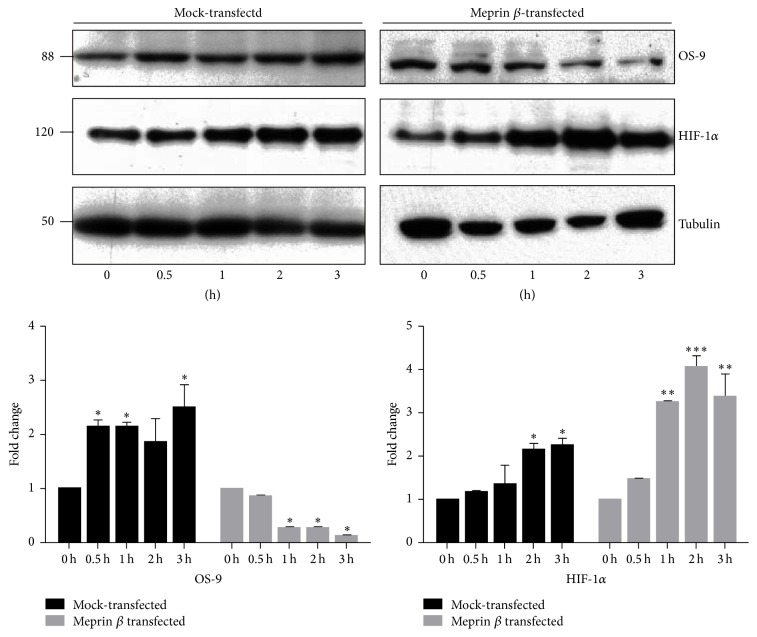
Immunoblots for OS-9 and HIF-1*α* in MDCK cells exposed to CoCl_2_. Meprin *β* transfected MDCK cells and mock transfected control MDCK cells were cultured in DMEM media supplemented with 10% FBS to 90% confluence, serum-starved overnight, and exposed to 125 *μ*M CoCl_2_ for 0–3 h. Western blot analysis and optic densitometry were used to quantify the levels of HIF-1*α* and OS-9 in the nuclear-enriched protein fractions. Tubulin was used as a loading control. The fold-change in protein levels was calculated relative to the 0 h levels. A time-dependent decrease in OS-9 levels was observed in meprin *β* transfected cells, but not in mock transfected controls cells. CoCl_2_ exposure induced an increase in nuclear levels of HIF-1*α* in both genotypes of MDCK cells. The relative OD was determined by the intensity of the protein relative to the intensity of the tubulin loading control as measured by spectrophotometry. The fold-change represents a comparison of the relative OD at each time point to the relative OD at 0 h. ^*∗*^ *P* ≤ 0.05; ^*∗∗*^ *P* ≤ 0.01; ^*∗∗∗*^ *P* ≤ 0.001.

**Figure 7:**
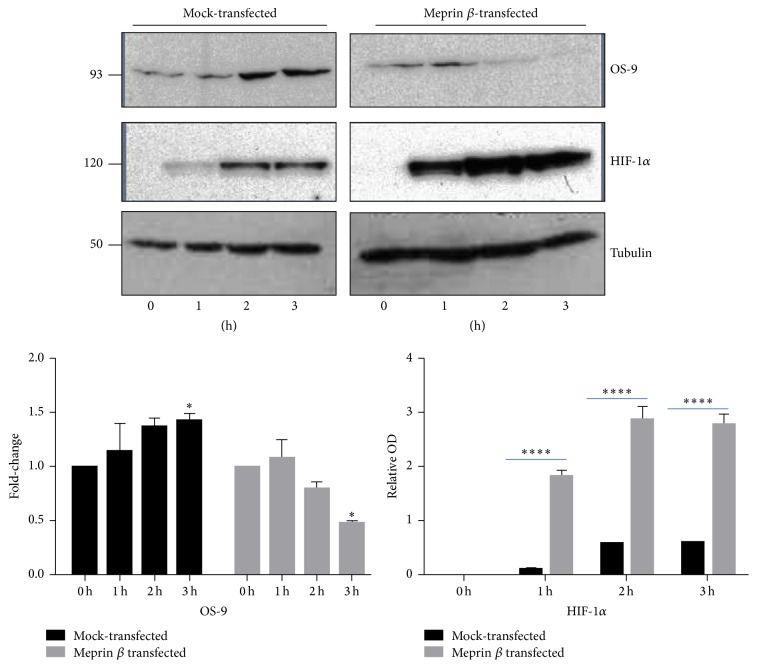
Immunoblots for OS-9 and HIF-1*α* in meprin *β* transfected HEK293 cells exposed to CoCl_2_. HEK293 cells were cultured in MEM with 10% FBS to 90% confluence, serum-starved overnight, and exposed to 125 *μ*M CoCl_2_ for 0–3 h. Proteins were extracted and fractionated into cytosolic- and nuclear-enriched fractions. Western blot analysis and optic densitometry were used to evaluate the levels of OS-9 and HIF-1*α* in the nuclear-enriched protein fractions. Tubulin was used as a loading control. CoCl_2_ exposure resulted in a time-dependent decrease in OS-9 levels in the meprin *β* transfected cells but not in the mock transfected controls. Stabilization of HIF-1*α* was evidenced by increased nuclear protein levels in all the cells exposed to CoCl_2_. The relative OD was determined by the intensity of the protein relative to the intensity of the loading control as measured by spectrophotometry. The fold-change represents a comparison of the relative OD at each time point to the relative OD at 0 h. ^*∗*^ *P* ≤ 0.05; ^*∗∗∗∗*^ *P* ≤ 0.0001.

**Figure 8:**
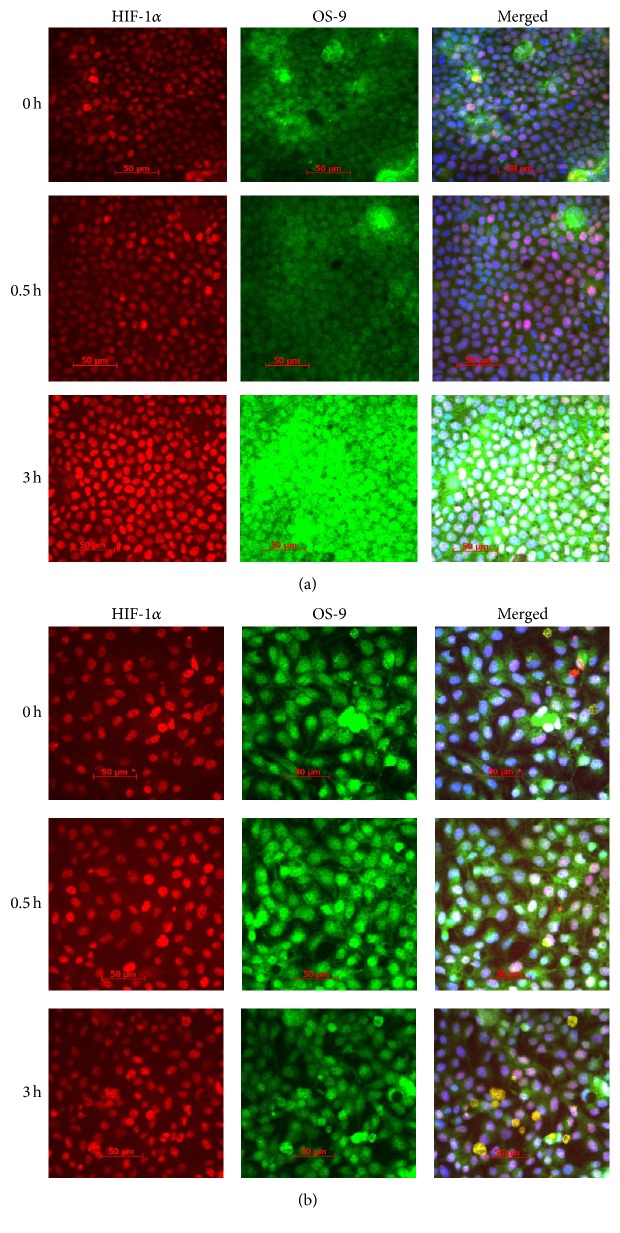
Immunofluorescence staining for HIF-1*α* (red) and OS-9 (green) in MDCK cells following exposure to CoCl_2_. Mock transfected control (a) and meprin *β* transfected MDCK cells (b) were cultured in chamber slides to 80–90% confluence. The cells were serum-starved overnight, exposed to 125 *μ*M CoCl_2_ and fixed in 1% paraformaldehyde. Anti-HIF-1*α* and anti-OS-9 antibodies were used to evaluate localization of HIF-1*α* and OS-9 in the cells by confocal microscopy. There was a time-dependent increase in nuclear levels of HIF-1*α* in both genotypes of cells, confirming stabilization of HIF-1*α* in the nucleus. The cytosolic levels OS-9 increased in a time-dependent manner in the nontransfected MDCK cells following exposure to CoCl_2_ (a). This increase was not as dramatic in meprin *β* transfected MDCK cells (b).

To evaluate* in vitro* interactions between meprin B and OS-9, we used meprin *β* transfected Madin-Darby canine kidney (MDCK) cells and human embryonic kidney (HEK293) cells. We first used Western blot analysis to determine localization of OS-9 in MDCK cells exposed to CoCl_2_. Our data showed that OS-9 is predominantly present in the nuclear-enriched protein fraction (Figure 5(a)). Western blot analysis also confirmed expression of meprin B in the meprin *β* transfected MDCK (Figure 5(b)) and HEK293 cells (data not shown). Exposure of the cells to the hypoxia mimic, CoCl_2_, resulted in a time-depended stabilization of nuclear HIF-1*α* leading to increased levels of HIF-1*α* in both MDCK (Figure 6) and HEK293 (Figure 7) cells. We then determined changes in the nuclear levels of OS-9 in MDCK and HEK293 following exposure to CoCl_2_. In mock transfected cells, there was a 1.5–2-fold increase in OS-9 levels following CoCl_2_ treatment. This increase was observed at 0.5 h after CoCl_2_ exposure and was sustained through the 3 h time course. In meprin *β* transfected cells on the other hand, the levels of OS-9 significantly decreased (50–75% decrease; *P* ≤ 0.001) following exposure to CoCl_2_ (Figures 6 and 7). Immunofluorescence staining and confocal microscopy also showed that both the nuclear and cytosolic levels of OS-9 increased following exposure to CoCl_2_ in nontransfected MDCK cells (Figure 8(a)). However, this increase was not observed in meprin *β* transfected cells, where in contrast the OS-9 levels decreased in a time-dependent manner following exposure to CoCl_2_ (Figure 8(b)). An equally important observation was the correlation between meprin B degradation of OS-9 and the relative levels for nuclear HIF-1*α* (Figures 6 and 7). In mock transfected MDCK cells, we observed a more modest increase in HIF-1*α* (~2-fold at 2 h). However for meprin *β* transfected MDCK cells, the fold-change in HIF-1*α* was significantly higher (~3 fold at 2 h) (*P* ≤ 0.0001). MDCK cells also had higher basal levels of HIF-1*α* when compared to HEK293 cells, for which HIF-1*α* levels were below detectable levels. The relative quantities for HIF-1*α* were significantly higher in meprin *β* transfected HEK293 cells when compared to the mock transfected control cells.

## 4. Discussion

Kidneys are highly susceptible to hypoxic conditions. Ischemia is the leading cause of hypoxia in the kidney. Acute kidney injury caused by ischemia/reperfusion (IR) occurs in nearly 5% of hospitalized patients. The reperfusion phase of IR is associated with oxidative stress, cellular dysfunction, and altered signal transduction. In IR-induced renal injury, disruption in the cytoskeleton leads to loss of the brush-border, a breakdown of cell junctions, and the incorrect relocalization of sodium-potassium ATPases from the basal surface to the apical surface [22]. Newly freed intracellular molecules of calcium activate proteases and phospholipases. This reperfusion event has been shown to be the causative agent of oxidative injury to tubular cells [23]. However, specific proteases that are activated in IR and their substrates are not fully understood. Meprins are metalloprotease that are abundantly expressed in the brush-border membranes (BBMs) of proximal kidney tubules. Changes in the level of expression and localization of meprins have been associated with the pathology of IR-induced kidney injury in mice and rats [1, 10, 24, 25]. While meprins are normally localized in the BBM of proximal tubules, redistribution of meprins to the cytosol and basolateral compartments is observed in IR [1, 4, 24]. This redistribution brings meprins in close proximity with protein targets in these compartments, which include mediators of the hypoxia response. The cells response to hypoxia is primarily mediated by hypoxia inducible factors (HIFs), which consist of two subunits, an oxygen-regulated *α* subunit and a constitutively expressed *β* subunit [26]. Under normoxia conditions, HIF-prolyl-hydroxylase promotes degradation of HIF*α*. However, hypoxia inhibits HIF-prolyl-hydroxylases, resulting in rapid accumulation of HIF*α*, which then binds to HIF*β* to form a heterodimer. The HIF*αβ* dimers translocate into the nucleus where they activate genes that promote adaptation to hypoxia by counteracting oxidative stress and improving cell survival. In the kidney, HIF-1*α* is expressed in tubular cells while HIF-2*α* is predominantly in peritubular cells, renal interstitial fibroblast cells, and endothelia cells. While HIFs are central in mediating the response to hypoxia, multiple mechanisms are involved in the pathophysiology of hypoxia-induced renal injury.

Osteosarcoma amplified 9 (OS-9), an endoplasmic reticulum (ER) lectin, is a protein coding gene located on 12q13 chromosome of humans. OS-9 is ubiquitously expressed in human tissues and is overly expressed in osteosarcomas [27]. OS-9 is part of the endoplasmic reticulum-associated degradation (ERAD) machinery and ERAD substrates and aids in the transfer of misfolded proteins [28]. Using RNAi, OS-9 was shown to be required for efficient ubiquitination of glycosylated ERAD substrates. There is increasing evidence that OS-9 is a critical component of the oxygen-signaling that upregulates HIF-1*α* [16]. OS-9 negatively regulates HIF-1*α* by binding to the HIF-1*α* and prolyl-hydroxylases (PHDs) promoting degradation of one of its subunits [16]. The presence of OS-9 promotes interaction of HIF-1*α* with prolyl-hydroxylase domain proteins PHD2 or PHD3, leading to hydroxylation and Von Hippel-Lindau (VHL) binding. By promoting degradation of HIF-1*α*, OS-9 could potentially modulate the hypoxia response and is a key player in the pathophysiology of renal IR. Studies using a yeast two-hybrid system showed that meprin *β* interacts with OS-9 [15]. However, it was not known whether meprins are capable of cleaving OS-9 and what the significance of such cleavage would be. The present study evaluated the interaction between meprin metalloproteases and OS-9* in vivo *and* in vitro*. Our data shows that meprin metalloproteases are capable of proteolytically processing OS-9* in vitro* and* in vivo*. Coincubation of meprins with kidney proteins from meprin *αβ*KO mice (which lack endogenous meprins) and purified OS-9 confirmed that the OS-9 cleavage was meprin B-specific and was inhibited by the meprin inhibitor, actinonin, at molar concentrations previously demonstrated to inhibit meprin activity [4]. To the best of our knowledge this is the first study to demonstrate that OS-9 is a meprin substrate. Previous studies have demonstrated differences in substrate and cleavage site preferences for the meprin protein isoforms, with meprin A selecting a variety of small and hydrophilic amino acids (e.g., proline residues) while meprin B prefers acidic residues (e.g., Asp/Glu-N peptidase) [29]. The present data further shows that hypoxia could induce meprin B degradation of OS-9* in vitro* and* in vivo*. A distinct 60 kDa OS-9 fragment was observed in the cytosolic kidney proteins from wild-type mice subjected to IR-induced renal injury, but not in samples from meprin *αβ*KO mice counterparts (which lack endogenous meprins). Interestingly, we did not observe accumulation of the 60 kDa OS-9 fragment when meprin B was incubated with purified OS-9. This could suggest that while meprin B cleaves OS-9, other factors present in the kidney tissue could play a role in stabilizing the cleavage products. Degradation of OS-9 was similarly observed in meprin *β* transfected MDCK and HEK293 cells exposed to the hypoxia mimic, CoCl_2_, suggesting that hypoxia enhances meprin degradation of OS-9. Studies that identify the meprin cleavage sites on OS-9 are thus needed to determine the functional implications of the meprin-OS-9 cleavage. Because OS-9 is believed to shuttle the hypoxia inducible factor (HIF) between the nucleus and ER, degradation would negatively impact this process. Using immunofluorescence staining and confocal microscopy, we further showed that, in nontransected kidney cell lines, there was a time-dependent redistribution of OS-9 from the nucleus to the cytosol over a 3 h period following exposure to CoCl_2_. However, in meprin *β* transfected cells, the hypoxia-induced cytosolic accumulation of OS-9 did not occur. Taken together, the data suggest that meprin B is partly responsible for the* in vivo* OS-9 fragmentation and that meprin B degradation of OS-9 blocks the cytosolic accumulation of OS-9 in the MDCK and HEK293 cells. The current data thus demonstrates a link between the previously reported interaction between OS-9 and meprin *β* to the pathology of acute kidney injury induced by IR. Indeed, the fold-change in nuclear HIF-1*α* levels was significantly higher in meprin *β* expressing cells under hypoxic conditions than in the nontransfected cells.

## 5. Conclusion

Previous work from our lab has shown that meprins are capable of proteolytically processing/degrading cytoskeletal kidney proteins, for example, actin and villin [4], the catalytic subunit of protein kinase A (PKA C) [7], and protein kinase C (PKC) [30], all in an isoform-specific manner. Furthermore, IR-induced acute kidney injury resulted in meprin-associated changes in PKA C profiles in mouse kidney tubules, leading us to conclude that the PKA signaling pathway is involved in the hypoxia response. Other known kidney meprin substrates include extracellular matrix (ECM) proteins [9–12], tight junction proteins, for example, E-cadherin and occludin [5, 31], and proinflammatory cytokines, for example, IL-1, IL-6, and IL-18 [21, 32, 33]. Of all the meprin substrates identified to date, OS-9 provides the most direct link to the hypoxia response and provides new insights on how meprins modulate tubular injury in IR. The HIF-1*α*/OS-9 interaction also links the hypoxia response to mitochondrial energy expenditure and reactive oxygen species (ROS) formation. This is supported by previous studies which showed upregulation of OS-9 in response to ER stress [28]. The present study and previous work from our group have provided evidence that meprins modulate the pathophysiology of IR-induced kidney injury via multiple mechanisms. Further investigations are needed in elucidating these mechanisms because the knowledge is important in development of therapeutic targets for acute kidney injury.
